# Multimodal imaging of angioid streaks

**DOI:** 10.3205/oc000165

**Published:** 2020-08-26

**Authors:** Sugandha Goel, Isha Gupta, Samarth Mishra, Barun Garg, Kumar Saurabh, Rupak Roy

**Affiliations:** 1 Aditya Birla Sankara Nethralaya, Kolkata, India

**Keywords:** angioid streaks, multicolor imaging, comet lesions

## Abstract

Angioid streaks (AS) are irregular crack-like dehiscences in Bruch’s membrane that are often associated with atrophic degeneration of the overlying retinal pigment epithelium. We herein report multimodal imaging of AS. Multicolor imaging highlighted AS in dark orange color. AS were better visualized in infrared reflectance as compared to green reflectance and blue reflectance. Peau d’orange appearance was seen as alternating dark and bright patches on color fundus photography with corresponding hyporeflective and hyperreflective patches on infrared reflectance. Comet lesions showed increased signal on infrared reflectance and hyperautofluorescence. Multicolor imaging is a non-invasive imaging modality which helps in clearly delineating these lesions.

## Introduction

Angioid streaks (AS) are irregular, bilateral, dark red to gray lines under the retina, radiating from the optic disc head in a spider-web configuration [1]. Knapp first coined the term “AS” because their appearance suggested a vascular origin [2]. Histologically, they are crack-like breaks in brittle, thickened, and calcified Bruch membrane (BM), and they may be associated with atrophy of the overlying retinal pigment epithelium (RPE) and the choriocapillaris layer [3]. AS can occur in isolation, or – most frequently – in association with pseudoxanthoma elasticum. Rarely it can occur in association with other systemic diseases such as Paget’s disease, sickle cell trait disease, and thalassemias [4]. Multicolor imaging (MC) is a non-invasive imaging modality available in the Spectralis platform. Utility of MC has been described in various disorders like diabetic retinopathy, retinal vein occlusion, and age-related macular degeneration [5]. We herein discuss MC signatures in a case of AS which has not been reported in the literature yet.

## Case descriptions

**Figure 1:**
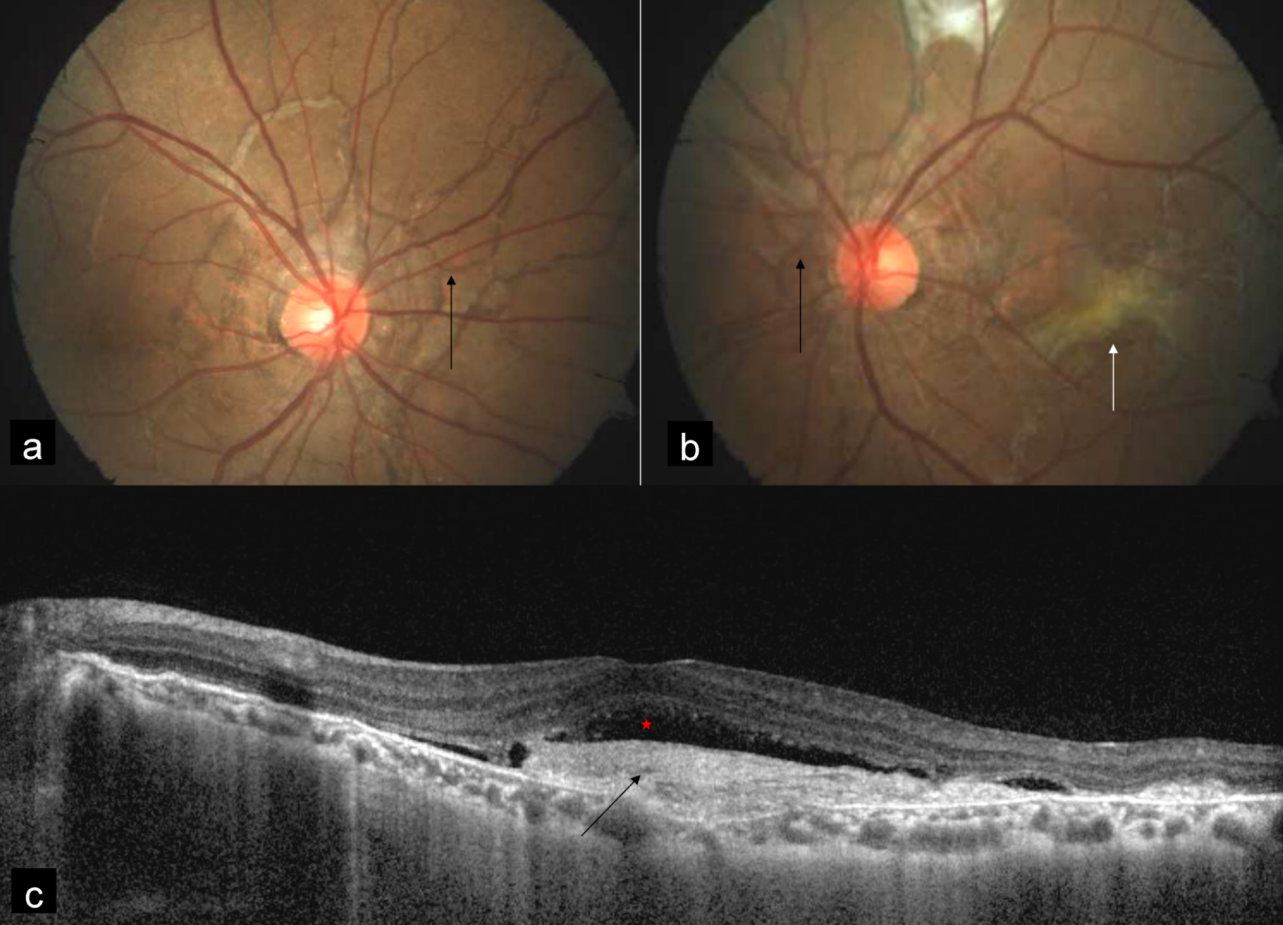
Color fundus photograph (CFP) showed angioid streaks (AS) in the right eye (a) and the left eye (b) (black arrows), and choroidal neovascular membrane (CNV) (white arrow) in the left eye (b). Spectral domain optical coherence tomography SD-OCT (c) showed CNV (black arrow) with subretinal fluid (red star).

**Figure 2:**
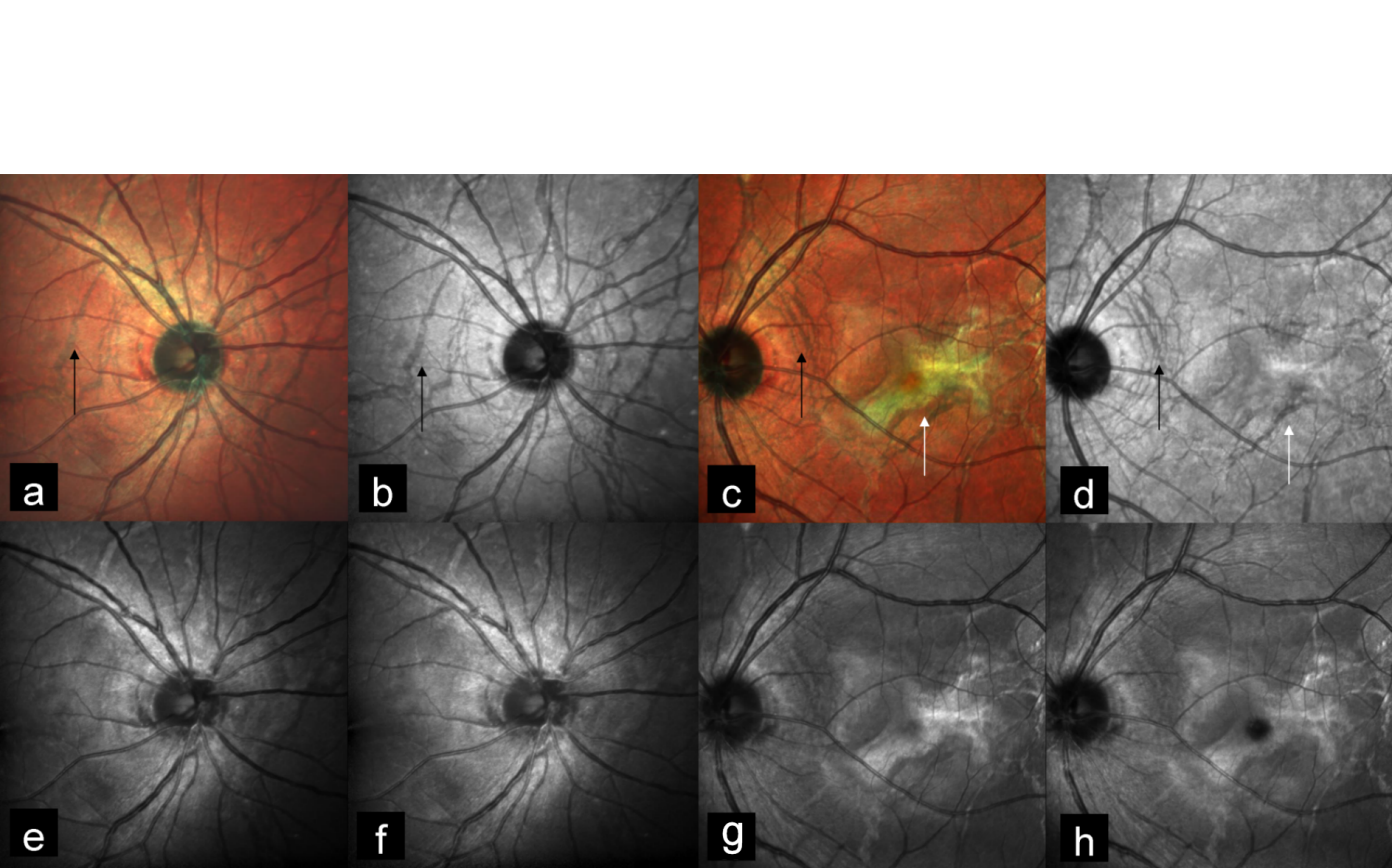
Multicolor imaging (MC) of both eyes highlighted AS in dark orange color (a, c) (black arrows). AS were better visualized in infrared reflectance (IR) (b, d) (black arrows) as compared to green reflectance (GR) (e, g) and blue reflectance (BR) (f, h). MC of the left eye showed CNV in greenish color (c) (white arrow) with corresponding IR (d), GR (g) and BR (h) images.

A 38-year-old man presented with diminution of vision in the left eye since 5 days. Best corrected visual acuity (BCVA) was 20/20 and 20/50 in the right and the left eye, respectively. MC was performed using confocal scanning laser ophthalmoscope (cSLO) with Heidelberg Spectralis HRA-OCT (Heidelberg Engineering, Dossenheim, Germany) with a scanning field of 30°. Color fundus photography (CFP) was done using FF 450 plus fundus camera (Carl Zeiss Meditec, Germany). CFP showed bilateral AS (Figure 1a, b (Fig. 1)) and choroidal neovascular membrane (CNV) in the left eye (Figure 1b (Fig. 1)). Spectral domain optical coherence tomography (SD-OCT) showed CNV with subretinal fluid in the left eye (Figure 1c (Fig. 1)). MC of both eyes (Figure 2a, c (Fig. 2)) highlighted AS in dark orange color. AS were better visualized in infrared reflectance (IR) (Figure 2b, d (Fig. 2)) as compared to green reflectance (GR) (Figure 2e, g (Fig. 2)) and blue reflectance (BR) (Figure 2f, h (Fig. 2)). MC of the left eye also showed CNV in greenish color (Figure 2c (Fig. 2)).

**Figure 3:**
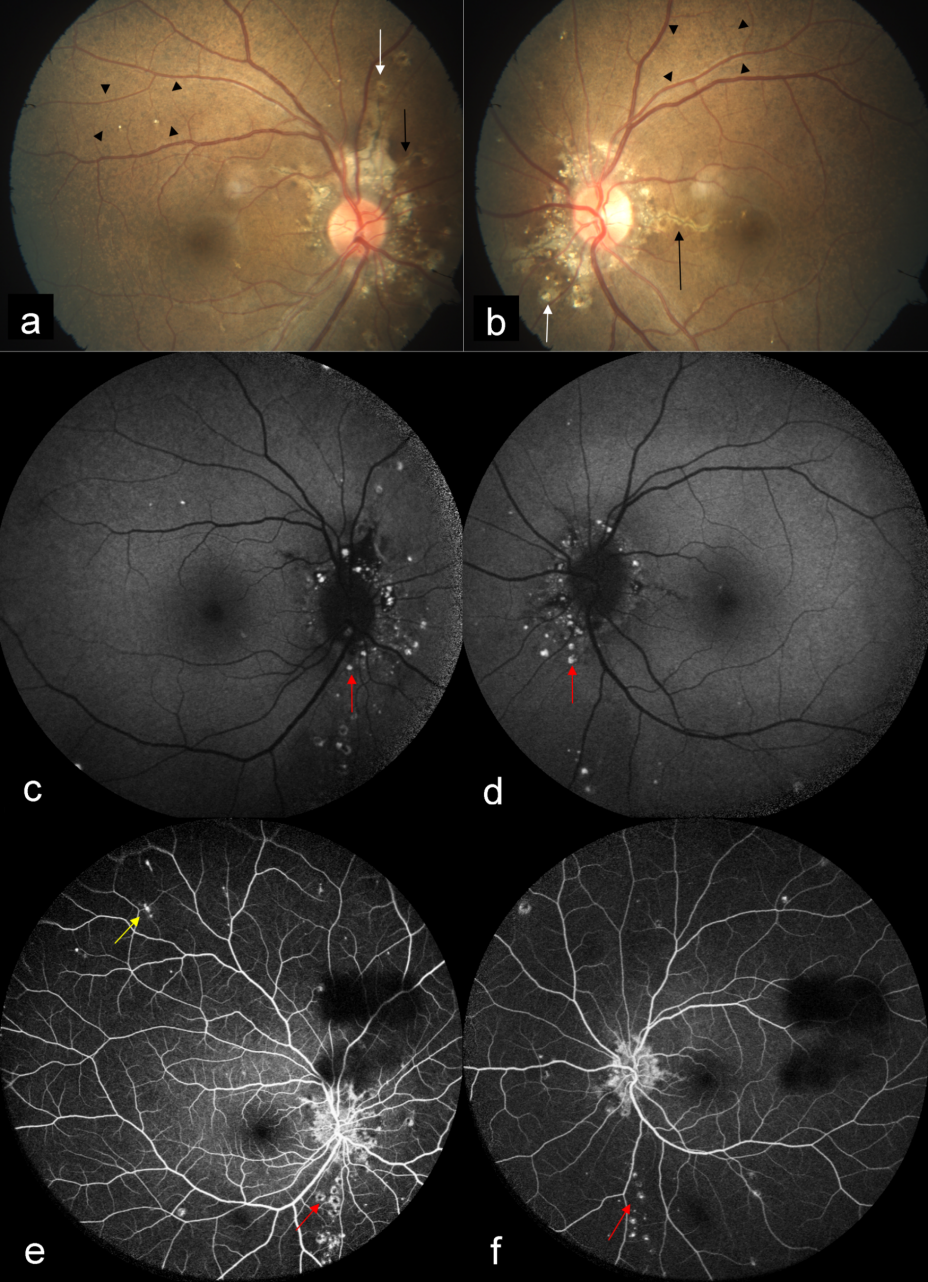
CFP (a, b) showed bilateral AS (black arrows) with peau d’orange as alternating dark and bright patches (arrow heads). Subretinal crystalline bodies and round white lesions without tails were seen surrounding the optic disc, suggestive of atypical drusen (white arrows). Spots of chorioretinal atrophy with variable size and “punched-out” appearance were seen in mid-periphery, suggestive of comet lesions. Comet lesions of both eyes showed a focal increased fundus autofluorescence (c, d) (red arrows) and a hyperfluorescence on late phase fundus fluorescein angiography (e and f) (red arrows). The associated comet tail that usually leaves the focal atrophy toward the posterior pole could be seen as hyperfluorescent in the right eye (e) (yellow arrow).

**Figure 4:**
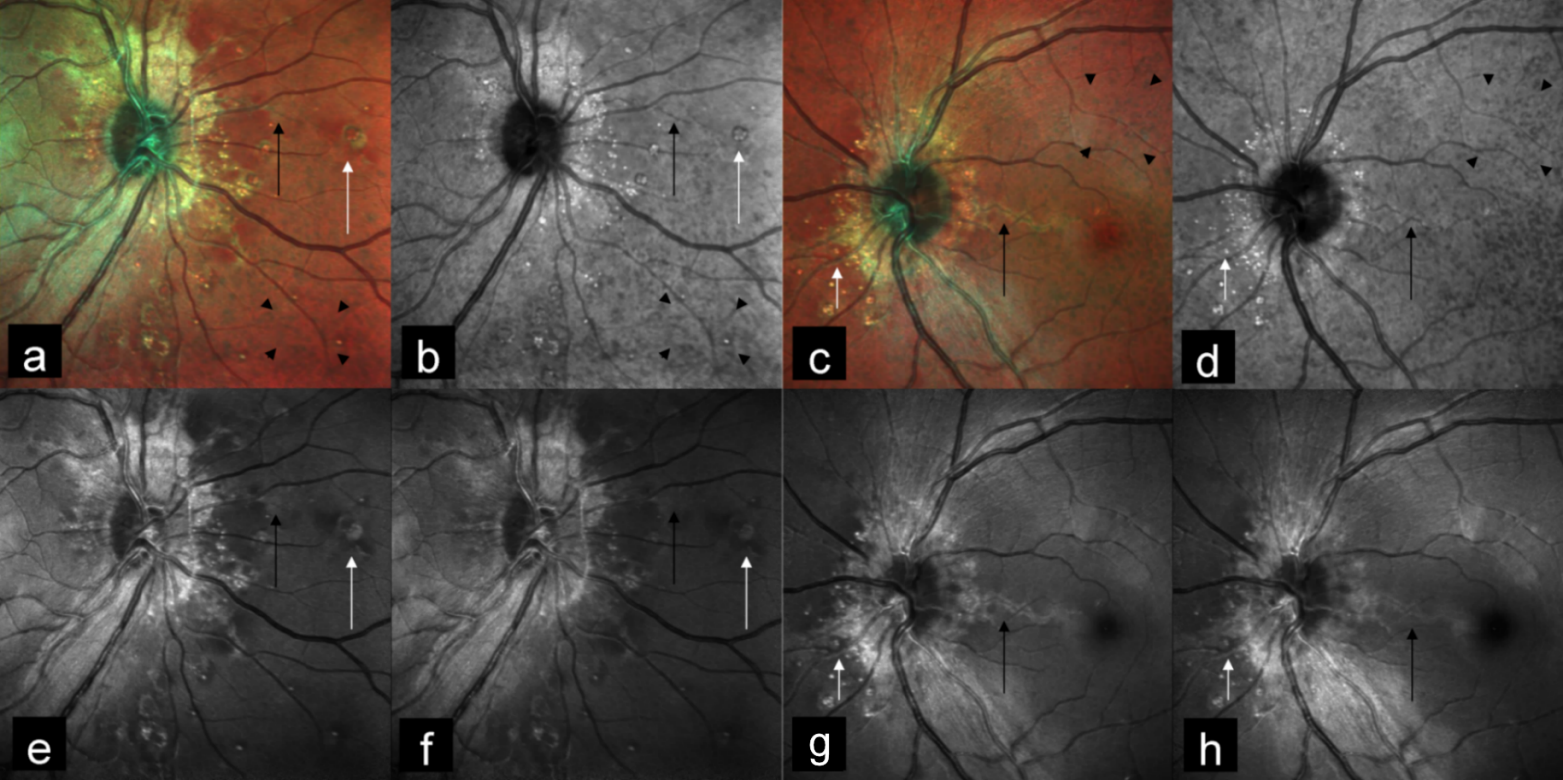
MC (a, c) of both eyes highlighted AS (black arrows), peau d’orange (arrow heads) and comet lesions (white arrows) clearly. AS was better visualized on IR (b, d) (black arrows). MC (a, c) highlighted peau d’orange in greenish color (arrow heads) which was seen as hyporeflective and hyperreflective patches on IR (arrow heads) (b, d). Comet lesions showed increased signal on IR (b, d), GR (e, g), and BR (f, h) (white arrows).

**Figure 5:**
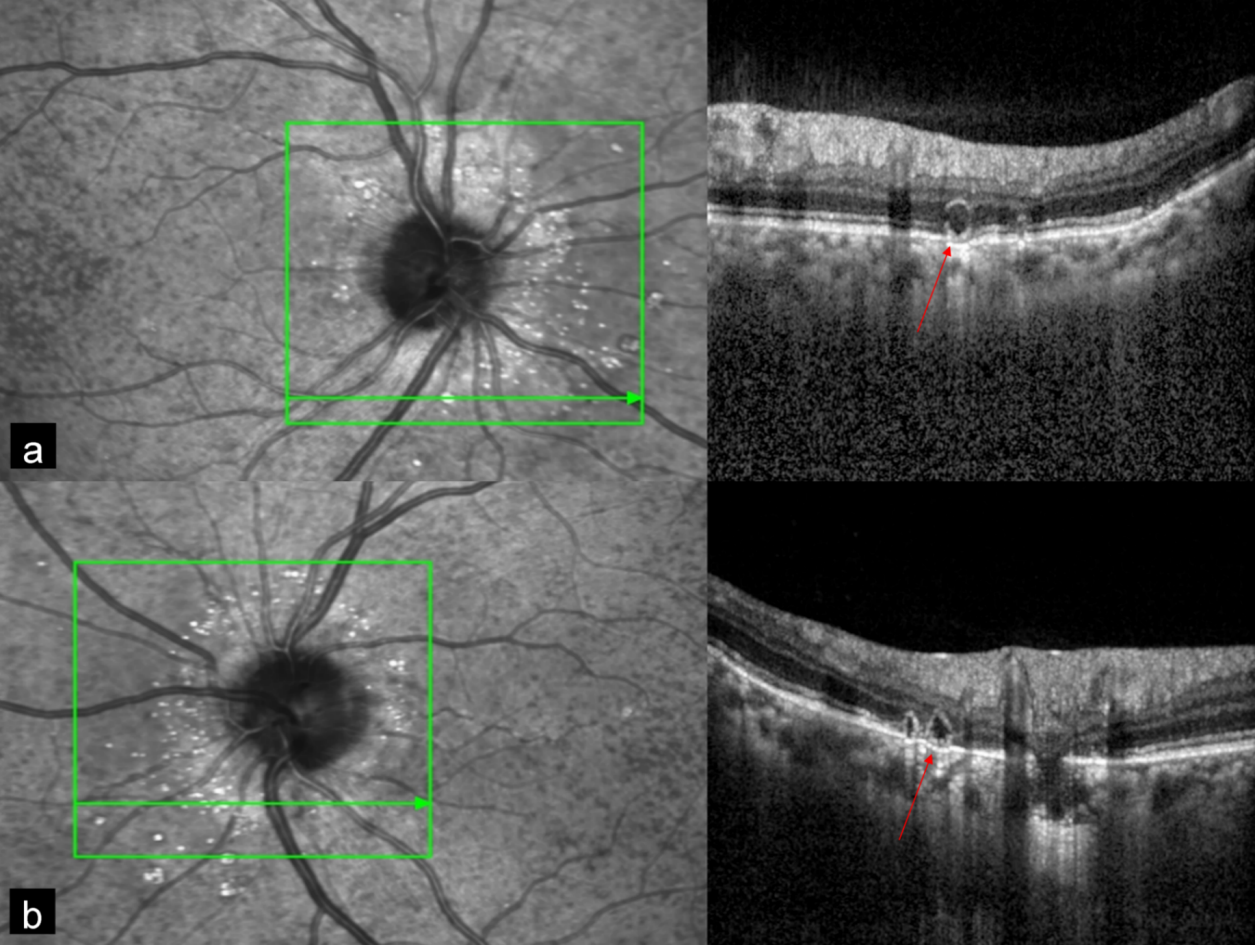
SD-OCT of the right eye (a) and the left eye (b) showed comet lesions as irregular hyporeflective spaces with hyperreflective inner border and focal debris like deposits above the retinal pigment epithelium (red arrows).

A 24-year-old woman came to our hospital for a routine eye check-up. BCVA was 20/20 in both eyes. CFP showed bilateral AS with peau d’orange appearance. Subretinal crystalline bodies and round white lesions without tails were seen surrounding the optic disc, suggestive of atypical drusen. Spots of chorioretinal atrophy with variable size and “punched-out” appearance were seen in mid-periphery, suggestive of comet lesions (Figure 3a, b (Fig. 3)). AS were seen as dark orange in color on MC (Figure 4a, c (Fig. 4)), and were better visualized in IR (Figure 4b, d (Fig. 4)) as compared to GR (Figure 4e, g (Fig. 4)) and BR (Figure 4f, h (Fig. 4)). Peau d’orange appearance was seen as alternating dark and bright patches on CFP (Figure 3a, b (Fig. 3)) with corresponding hyporeflective and hyperreflective patches on IR (Figure 4b, d (Fig. 4)). MC highlighted peau d’orange in greenish color (Figure 4a, c (Fig. 4)). Comet lesions showed increased signal on IR (Figure 4b, d (Fig. 4)), GR (Figure 4e, g (Fig. 4)), and BR (Figure 4f, h (Fig. 4)). On SD-OCT of both eyes (Figure 5a, 5b (Fig. 5)), comet lesions appeared as irregular hyporeflective spaces with hyperreflective inner border and focal debris like deposits above the RPE. Comet lesions of both eyes showed a focal increased fundus autofluorescence (FAF) (Figure 3c, d (Fig. 3)) and a hyperfluorescence on late-phase fundus fluorescein angiography (FFA) (Figure 3e, f (Fig. 3)). The associated comet tail that usually leaves the focal atrophy toward the posterior pole could be seen as hyperfluorescent on FFA in the right eye (Figure 3e (Fig. 3)). The patient was clinically diagnosed with pseudoxanthoma elasticum. She was referred for dermatology and cardiology opinion.

## Discussion

Peau d’orange, which gives the temporal macula an “orange peel” appearance, is seen in patients with pseudoxanthoma elasticum. The exact pathogenesis of this lesion is not clear [6]. Possible causes include pigment deposition abnormalities at the level of RPE, or calcification of BM [6], [7]. These lesions are seen as dark and light patches on CFP with corresponding hyporeflective and hyperreflective patches on IR. They are seen as greenish in color on MC due to scattering of light from an irregular thickening of the RPE. They were better visualized in IR as compared to GR and BR because of IR’s greater penetration depth. The superior visibility of peau d’orange with IR also locates the primary underlying disease at the level of RPE.

Comet lesions are small, roundish chorioretinal atrophies observed as white bodies in the mid-periphery of the fundus or closer to the optic disc [8]. They may present with a tail pointing toward the optic disk, thus the term „comet tail lesions“. At times, a collection of comets can be observed, creating an aspect of comet rain [6]. The pathogenesis of these lesions is unclear. They appear as irregular hyporeflective spaces with hyperreflective inner border and focal debris like deposits above the RPE, as seen in our second patient [8]. Multiple comet lesions could coalesce into a single lesion and could have full-thickness chorioretinal extension. They show increased signal on IR, but may be seen on BR and GR in cases of full-thickness chorioretinal degeneration, as seen in our second patient. They typically show a focal increased FAF. Whether this hyperautofluorescence is caused due to lipofuscin, or the presence of calcification, or its combination, is not yet clear. Comet lesions show hyperfluorescence, and associated comet tail could also be seen as hyperfluorescent on FFA. Subretinal crystalline bodies and round white lesions without tails in pseudoxanthoma elasticum have by some authors been described as atypical drusen [9], and by others as salmon spots [10]. We also found similar lesions in our second case. These lesions and comet lesions probably share a similar histopathology.

Patients with AS are usually asymptomatic unless they develop complications like choroidal rupture and CNV [11]. CNV is the major cause of severe visual loss in patients with AS, occurring in 42% to 86% of patients during follow-up [12]. MC of the left eye in our first patient showed CNV in greenish color due to retinal elevation.

## Conclusion

MC is a non-invasive imaging modality which helps in clearly delineating comet lesions.

## Notes

### Competing interests

The authors declare that they have no competing interests.
